# The Views of Researchers on the Alcohol Industry's Involvement in Science: Findings from an Interview Study

**DOI:** 10.1159/000522603

**Published:** 2022-03-22

**Authors:** Jim McCambridge, Gemma Mitchell

**Affiliations:** Department of Health Sciences, University of York, York, UK

**Keywords:** Alcohol, Science, Alcohol industry, Bias, Conflict of interest

## Abstract

**Introduction:**

Debates in the peer-reviewed literature on alcohol industry involvement in science have been polarized, with the activities of the International Center on Alcohol Policies and industry provision of research funding being particularly contentious. We aimed to explore researchers' views on the nature of the debates and the issues raised.

**Methods:**

Qualitative interview study with experienced researchers working on alcohol policy-relevant topics across ten countries (*n* = 37). Thematic analysis of views articulated, supported where appropriate by accounts of how experiences informed particular perspectives.

**Results:**

The main finding is how much common ground there now is among participants, regardless of whether they had previously worked with industry organizations or received alcohol industry funding. Norms have changed and participants agree that the earlier debates were dysfunctional. Participants on all sides of these earlier debates experienced significant psychological burdens as a result of industry-related activity in alcohol research. These include reputational harms from working with industry organizations and/or receiving research funding, and harassment by industry for producing findings contrary to commercial interests. Key ongoing contentious issues include the extent to which conflicts of interest can or should be managed by individual researchers, and how distinct the alcohol industry is from other funders and other industries. Participant views on ways forward include improving the evidence-base underpinning the debates, and having collegiate discussions among researchers, including all strands of opinion and experience.

**Conclusions:**

This group of alcohol researchers shares more nuanced contemporary positions on issues relating to industry involvement in science than are reflected in the existing material in peer-reviewed journals. Almost all regard the alcohol industry's involvement in research as having been damaging.

## Introduction

There has been a high level of concern about alcohol industry involvement in science [1, 2, 3, 4] and in evidence-informed public health policy-making [5, 6, 7] for decades. Despite this, with notable exceptions (e.g., [4, 8, 9]), the research community has been slow to develop a tradition of the study of alcohol industry involvement in science. This may be partly because this has been a highly divisive subject, with some researchers working with industry actors, and others strongly opposed.

The study of the ways in which alcohol industry actors seek to influence policy, and use scientific evidence in so doing, has developed more extensively [10, 11, 12, 13, 14, 15, 16, 17]. For example, industry actors may intervene in peer-reviewed scientific journals to create artefacts that are useful in making policy claims [18] or operate on the fringes of science, producing reports that make claims about science which are subsequently used in attempts to influence policy [19]. Existing findings resonate strongly with the wider body of evidence available on the tobacco industry and other corporate actors' moulding of science to shape public policies on health and environmental issues [20, 21, 22, 23].

A systematic review analysed the content of concerns about alcohol industry involvement in science and the nature of the resulting debates [24]. That study found there were three sets of concerns articulated within debates in scientific journals. Firstly, to do with the motivations for industry involvement, being primarily a vehicle for the advancement of commercial rather than scientific or public health goals, despite claims to the contrary. The activities of the International Center on Alcohol Policies were prominent in this regard. Secondly, there were various ways in which bias could be introduced to the literature. Thirdly, industry actors tended to ignore or otherwise transgress basic norms involved in doing scientific work [24].

In contrast, there was a coherent set of views that opposed these concerns, and this perspective was held by a minority of those involved in the debates, often arguing that the concerns were too simplistic and/or moralistic [24]. There have been few formal studies building the evidence base in this area [24].

This is the first interview study of this subject and is necessarily preliminary. We have elsewhere reported on the ubiquity of alcohol industry involvement in research [25], experiences of early career industry research funding [26], and of working with industry organizations later in careers [27]. This report is concerned with the perspectives of alcohol researchers on the broader issues raised by alcohol industry involvement in science.

## Methods

This study received ethical approval from the University of York Department of Health Sciences Research Governance Committee. We drew extensively on a systematic review of researcher concerns about alcohol industry involvement in science [24], applying a qualitative approach, informed by the sociology of knowledge and science and technology studies, to explore the values and meanings researchers attach to their scientific work or “practice” [28, 29]. This thematic analysis examines views on the debates about working with industry in these contexts. It should be noted that the first author has been an active participant in discussions of this topic within the research community (e.g., [30, 31, 32]); the second author, who conducted all interviews, had no prior experience of working in this field and did not know the participants. Here, “alcohol industry” largely refers to transnational alcohol producers [33] due to their involvement in research, rather than smaller, retail businesses, though note that in some countries national trade associations including those representing retail are important.

In sampling and recruitment, we sought approximately equivalent proportions of experienced researchers working on alcohol policy-relevant topics who had or had not “worked with” the alcohol industry to any significant extent. This mainly drew on the first author's knowledge of the field, and we targeted health and social science researchers, rather than chemists for example. We invited 44 researchers to take part in the study, with an encouraging 37 researchers across ten countries accepting the invitation. It transpired that the majority of participants (*n* = 23/37) had direct personal experience of relationships with industry actors. The large majority of the participants were based in the UK (*n* = 17), the USA (*n* = 9), or Australia (*n* = 3) reflecting that most published alcohol research takes place in high-income Anglophone countries. Of the 37 participants, 12 were female and 25 were male, reflecting the gender disparity in alcohol research at senior levels. The resulting sample thus comprised scientists with careers that had progressed through promotion, research funding, and attaining senior roles to mid- or late-career stages or were formally retired. All participants gave informed consent prior to taking part in the study. We do not in any way claim the sample is representative of the alcohol research community, and do not generalize inferences beyond this group.

Semistructured interviews took place between March and July 2019 and were conducted by phone, video, or, least often, in-person. There were no obvious differences in data resulting from different modes of interview. Interviews ranged from 30 min to 2 h, usually lasting 60–75 min; all were audio-recorded and subsequently transcribed. Industry involvement in science can be an emotive subject, engendering challenging personal experiences in some cases, which were reflected in the interviews. These began by eliciting reflective accounts of the development of one's own career, exploring encounters with industry, and views about the issues raised in the literature.

After initial coding in NVivo of the entire dataset by the second author, who led on the generation of themes in the main wave of analysis using a form of reflexive thematic analysis [34, 35], the first author then led the thematic analysis of the data specifically on views examined here. The analysis situated the data in context of the findings from the systematic review on the topic [24] and was refined by both authors. We draw on accounts of experiences when they have been used by the participants to explain particular perspectives. The analysis process was concerned with the manifest content of the views articulated and did not seek to critically interrogate them. We thus do not analyse what the participants are doing in the interviews, other than sharing their views. We have removed all identifying information about the participants from the quotes provided below and use participant numbers to protect researcher anonymity. Participants were offered a range of consent options to choose from regarding direct quotation, and we have directly quoted 13, eight of whom had received funding from, or worked with, industry actors.

## Results

The main finding, and one which is somewhat surprising in view of the highly polarized nature of published debates on alcohol industry involvement in science, is how much common ground there is among participants, regardless of their past or present relationships with the alcohol industry. The extensive common ground will first be presented, prior to consideration of the ways in which differences open up, and participant suggestions for possible ways forward.

Those who have chosen to work with industry organizations or seek industry funding have largely sceptical attitudes toward industry and are very careful in their decision-making. Such perspectives developed over time as a result of experiences, which are mostly, but not entirely, negative. The views of those who have not worked with industry in any way are also heterogeneous in various respects within the largely shared common ground. The highly polarized nature of the debate in the literature is largely organized around binary positions on the legitimacy of receipt of industry funding. In many ways, alcohol researchers have much more nuanced positions than are reflected in that somewhat narrowly circumscribed debate.

### The Prior Debate Was Dysfunctional

There is a clear recognition among participants of the corrosive nature of earlier debates on this subject, with differences articulated on the degree to which this was the case. For one researcher: *It's created dissention within the scientific community among well-meaning people.* P4

While for another: *There is a nastiness about the discussion sometimes*. P14

This had the effect of undermining the collegiate basis for discussion of what were accepted as challenging issues, particularly felt by those who have worked with or received funding from industry. This problem was also acknowledged by those without such connections: *What we're doing is we're going to people saying you have accepted money, therefore you are tainted, and that's probably not a good way to start a conversation*. P7

This was vividly illustrated by one researcher: *It made me feel that I was being told that I must not have any contact of any kind with people in the industry… and I decided that I was [laughs] definitely going to have contact with them so it actually pushed me in the other direction.* P22

Moralization of the issues is regarded as self-defeating also for those who do have concerns about industry involvement in the research community. It is also viewed as detrimental to any sense of shared purpose, which is intrinsic to advances in research. As one researcher who had worked with an industry organization suggested: *As researchers, we have to stick together, we have to respect each other, and causing a divide between researchers probably does nothing but help the industry.* P28

The accumulated learning about the need for careful discussion within the community extended to informal conversations. One participant with long involvement with industry actors reflected that useful conversations were those which simply facilitated their own reflections in non-threatening and non-stigmatizing ways.

A key issue inhibiting discussion in the community was the weakness of existing evidence on industry involvement in science, resulting from the limited extent of prior study. This was seen as difficult work to have funded, and the value of stronger evidence in this area seen as clear: *The problem is that many of the publications about industry involvement are much more driven by a sort of view than driven by evidence and, therefore, if we want to change that we need much stronger evidence*. P15

### How Did It Come to This?

Although there was agreement that scarcity of research funding had impacted both experiences of, and debates on industry, perceptions of this issue operated in somewhat distinct ways among those who had avoided any relationship with industry. Not only did it limit investigations into industry activities, many participants saw this as providing an opportunity for industry actors to plug gaps in research funding systems. This allowed opportunities to build relationships with researchers, particularly those who may find it more difficult to obtain funding elsewhere. There were seen to be many possible reasons implicated, including the resources available in low- and middle-income countries, the quality of the research, how interested industry might be in the topic, and institutional and disciplinary affiliations making access to funding more difficult.

Many of these same issues were prominent for those participants who sought funding from industry early in their careers. For the small number of participants in receipt of industry funding only later in their careers, the system of soft money project grant funding led them to think about a mixed economy of funding sources including industry money. According to one: *I needed some work, I needed some coverage too, so that was another consideration -what are my prospects for getting some new grants funded from [public funder]? And, I thought, you know, maybe I should hedge my bets here a little bit and, worst case scenario, I'm not going to get enough grants funded.* P32

This inherently precarious situation created risks of dependence on industry funding that participants were well aware of, and reported having to manage carefully. The situation posed obvious dilemmas for participants, and so it was widely seen as important to articulate the issues more strongly to funders. In the absence of such measures, and even if they were applied in high-income countries, there was a shared sense that these issues will continue to play out differently in low- and middle-income countries.

### The Times They Are A-Changing

Almost all participants were of the view that the debates about alcohol industry funding and working with the industry had moved on in important ways in recent years. There were varying perceptions of the degree of change, and different attributions made about reasons for it. In some cases, this was seen as a consequence of earlier attention within the alcohol field, and others cited broader influences. Reputational harms from being seen to be too close to industry actors were highly salient for some. Those who have sought industry funding or worked with industry organizations were keenly aware of this issue, and they particularly articulated a sense that the atmosphere had shifted in recent years: *Regardless of what one thinks about the issues, the balance of decision-making is now different; the hostile environment that's being created around it. So why would you risk it as an early career person?* P28

This converged with disappointing experiences in the activities with which they had been involved, bringing their views closer to those who had no such involvement with industry. Alcohol research is also not conducted in isolation from other public health, biomedical, and social sciences, and the awareness of wider developments was also cited as influential: *I think the dial has moved on what's acceptable and what isn't. We're much closer to the tobacco world now where you just can't take industry funding… I just have sympathy for colleagues who are in a difficult position*. P5

Participants recognize that, despite changes to the way industry funds research in recent years, there are resources available for the conduct of research that may not otherwise be possible to access. Fewer colleagues still nurture hopes that there remain possible gains to public health from working with industry. The content of their aspirations appears to be changing, however, with a small number of participants identifying product reformulation (reducing product alcohol content) as a key possible benefit from working with industry, exerting pressure for change. There is no longer any mood of optimism among the majority of those with experience of working with industry that it will somehow become a public health actor.

### Key Points of Contention on Conflict of Interest

There remain important differences of opinion between those who have and have not worked with industry, and also within these groups. Those who have chosen not to work with industry emphasize to a greater degree what they regard as inherent and ungoverned conflicts of interests arising out of industry involvement in science, and indeed there is a widespread view in that group that this is *the* key issue. Those who have worked with industry know well the risks, and the burden this creates, but view this, at least in principle, as manageable by the individual. This difference plays out sharply in perceptions of the risks of bias and how they may be managed. As someone who worked with industry stated: *There are ways in which the choice of analysis, the choice of post-hoc analysis, can be subtly influenced. And if your funding is depending on a certain type of resource, then you have to be extremely careful that unconscious − well, subconscious − biases aren't creeping in*. P20

Yet, for participants who have avoided industry, any emphasis on the individual ignores the systemic nature of the problems associated with research sponsorship across a wide range of industries: *Despite all the evidence that this does impact on what you find, that [industry involvement] damage[s] the science, they can still say it's not affecting me. And if people can delude themselves like that how can I possibly trust their ability to come to rational conclusions*? P1

There are differing views about the extent to which the tobacco experience in particular is relevant to alcohol. Similarly, there are differing assessments of the threats posed by industry funding effects and the interests of other, non-industry, funders. One group tends more toward a position of equivalence in respect of the potential biasing effects of all funders, whereas the other group is especially concerned about industry as a source of bias.

The majority of participants regarded it as possible to reduce the divergence of views by prioritizing these issues for evidence generation. This was seen as needed not so much to heal old wounds, but out of a recognition that the issues were too important for the research community to ignore.

### Views on Ways Forward

Consensus among participants on the need for further change has multiple origins, prominently including a sense of frustration at the lack of progress in the application of the evidence in public health decision-making; at the limited funding available for research; from prior attempts to work with industry; and from the psychological burdens involved in ongoing work including harassment by industry for producing findings contrary to business interests. Individuals making decisions in isolation about such challenging issues were seen to involve obvious pitfalls and were made more burdensome by limited support from colleagues. Two key ideas that were widely articulated were the need to improve the evidence base and the benefits of collegiate discussion of both the evidence and the issues. The dilemmas and tensions felt as a result of the complexity of individual decision-making face everyone, but have been most widely problematic for those who have received industry research funding: *I do recognise and experience the tension that's there. Ideally we wouldn't do this. Ideally there would be enough independent funding, by that I mean disinterested funding*. P24

More acutely, another suggested: *It is kind of hard feeling like I'm doing something unethical perhaps, or at least that's how it's viewed by many of my colleagues.* P32

Interestingly, for different reasons, the smaller number of participants who have been attacked by industry, either as a result of studying the industry or producing findings that run contrary to business interests, endure similar kinds of burdens to those who have accepted industry funding. Their experiences suggest that the industry approach appears to be designed to make such work more difficult to do.

Collegiate discussions were regarded as having potential for producing guidance that was not moralizing, that benefitted from direct experiences of industry involvement in science, and which was clear about the risks involved. Importantly, all but four participants would advise or guide early career researchers away from working with industry, including most who had done so themselves. A small number who would give this guidance are nonetheless ambivalent about the broader issues. This is because whilst they articulate views on the damaging nature of industry involvement in research, they also share to a lesser extent some of the views endorsed by the four remaining participants; namely, on conflict of interest and the equivalence of bias resulting from other types of funders, as well as a concern about moralization.

If guidance is to be produced on these issues, the widest possible consensus was regarded as vital to obtain, without the content being meaningless. Such guidance should not be aimed only at individual researchers: for example, participants pointed out that industries have the capacity for influence in universities in ways far beyond the award of research grants. Hence, there was some discussion of institutional conflicts of interest and widespread agreement that: *If we really felt that we needed to do something about industry influence, I think it has to be at organisational levels.* P11

This means that the field's institutions, such as academic societies and journals, need also to be engaged, if any resulting guidance is to be effectively promoted and used by the wider alcohol research community. Participants also suggested there may also be useful lessons to be drawn from alcohol for wider research communities if progress can be made in reversing the damage done by industry involvement and moving the field forward together.

## Discussion

The main finding is that there is in many ways a shared understanding of the issues, even where different decisions were made about proximity to industry. The older debates in the journals were rancorous and may have delayed progress in understanding. Scarcity of research funding was and remains a key reason for industry success in entering the field. Notwithstanding a lack of progress in the study of the issues, norms have changed, though issues to do with conflict of interest remain deeply contentious. The experience of industry involvement in research has been damaging in different ways, and it should now be possible to discuss the emerging evidence and associated issues in a respectful and collegiate manner.

The views outlined here should be considered in the context of participant experiences of industry activity. We have reported elsewhere that participants who approached industry for funding early in their careers developed longstanding involvements in industry-related networks, despite the grants having no strings attached [26]. Those who began working with industry organizations later in their careers were typically disappointed that opportunities to make genuine contributions to research translation did not materialize [27]. Industry has had a ubiquitous presence in alcohol research such that researchers who decided to have nothing to do with industry throughout their careers were nonetheless confronted with decisions about how to manage encounters with industry [25]. All decisions were profoundly shaped by the broader contexts in which careers developed.

Before discussing study strengths and limitations, some reflection on our practice is appropriate. This study demonstrates there is enormous value in listening to researchers about the sensitive and challenging issues they face, as a fuller understanding of these issues may be attained. A limitation to be faced in reporting this study is that the material covered is both extremely important and all too rarely discussed, and this brief report can necessarily only open up this territory for further exploration rather than undertake a definitive study. The views presented here are strongly patterned by personal experiences, which the discussions explored confidentially and anonymously, and which have been valuable to elicit. The intention of the analysis was to seek to provide a fair summary of the content of the views expressed without unduly imposing our own perspectives on the data. We also cover the views of a large number of people on a complex subject, necessarily briefly. Unavoidably, indeed by design, the prior systematic review framed our approach to the study design and data analysis. Readers may judge for themselves how far the present findings add to the analysis therein and make a credible, trustworthy, and novel contribution.

Our sample includes participants interested and willing to discuss the role of the alcohol industry in science, thus a sense of shared purpose may be more likely in this group than more widely, where researchers may have different motives for working with the alcohol industry. This is important as the large majority of the research funded by industry is not concerned with directly policy-relevant areas e.g., chemistry [36]. Participants were largely based in high-income countries, and the thinking about the issues was regarded as ethnocentric by some. Further studies should be extended to include researchers in low-income countries and include early career as well as established researchers. There were, however, a variety of disciplines and research topic interests covered. The level of participation and range of views represented are encouraging and suggest the first author's prior work did not obstruct data collection. Indeed, a key strength of the study is the representation of views from participants who have current or previous connections to industry organizations. A key limitation may be the depth of the analysis possible on subjects relating to conflict of interest, which we suggest is particularly in need of further careful analysis given how contentious it remains.

It is clear that the narrow focus of published debates does not reflect the diversity of experiences and views, which this study demonstrates is possible to investigate in ways that advance understanding. Divisions in the research community have been based on a crude, indeed false, dichotomy (“for” or “against” industry). Instead, researchers on all sides of the debates have much in common. Most of them experience some form of psychological burden as a result of industry involvement in science and thus appreciate harm personally. Opposing views may be strongly held, and recognition of commonalities in experiences and shared interests provides a productive starting point for their exploration. Further evidence on the underlying issues will be important in managing what has often been an emotive subject. Attention should be paid to changes in the way alcohol companies provide funding to researchers in recent years, including the legacy of now-closed industry funding organizations. Improving the evidence on researchers' experiences of industry in different contexts, topics of interest, disciplines, and countries will also be helpful.

Two areas where views diverge are the extent to which conflicts of interest regarding industry can be managed by the individual, and relatedly the extent to which industry vested interests in outcomes are equivalent to those of other funders. For the former, existing attention to a range of industries [37, 38, 39, 40] suggests that these are systemic problems requiring collective solutions, and there are science policy issues at stake that industry seeks to influence [16, 41]. For the latter, industries are not the only funders who have vested interests in research outcomes and can influence its conduct [42, 43]. Both issues are amenable to further study.

The vast majority of participants agreed that meeting to discuss the issues in-person was important to move the debates forward. There may lurk, however, deeper causes of lack of agreement than have been uncovered in this preliminary study. There are also bigger institutional contexts to be borne in mind; universities and research funders have important roles to play, and it may be fruitful for researchers to consider ways in which explorations of the challenging issues raised here may find their way on to high-level agendas. There can be no place for industry representatives in future discussions among researchers, as this would stymie the process as industry involvement is regarded here as the fundamental source of the divisions in the research community. Such discussions would certainly need, however, to welcome those currently or historically involved with industry, and indeed to value their experience as a source of learning, making efforts to protect the collegiate nature of the discussions. There are burdens and complexities, nuance and angst, in the views held by participants and the interviews not infrequently involved heightened emotions. Nonetheless, regardless of varied experiences of industry involvement in research, most participants shared views in common. Our findings thus lead us to question whether deep divisions in the research community on this issue remain that cannot be resolved.

## Statement of Ethics

This study received ethical approval from the University of York Department of Health Sciences Research Governance Committee (HSRGC/2019/316/C). Written informed consent was obtained from participants.

## Conflict of Interest Statement

The authors have no conflicts of interest to declare.

## Funding Sources

This work was supported by a Wellcome Trust Award (200321/Z/15/Z) to the first author. The funder is not involved in the design of this study nor in the decision to publish this study. For the purpose of open access, the authors have applied a CC BY public copyright licence to any Author Accepted Manuscript version arising from this submission.

## Author Contributions

The first author led the detailed thematic analysis, with the second author revising with important intellectual content. The first author wrote the first draft, with both authors working on successive drafts. Both authors read and approved the final manuscript.

## Data Availability Statement

Given the sensitive nature of the data, and in line with the consent procedures, there are no data available for sharing.
